# Proposal of an obesity classification based on weight history: an official document by the Brazilian Society of Endocrinology and Metabolism (SBEM) and the Brazilian Society for the Study of Obesity and Metabolic Syndrome (ABESO)

**DOI:** 10.20945/2359-3997000000465

**Published:** 2022-04-19

**Authors:** Bruno Halpern, Marcio C. Mancini, Maria Edna de Melo, Rodrigo N. Lamounier, Rodrigo O. Moreira, Mario K. Carra, Theodore K. Kyle, Cintia Cercato, Cesar Luiz Boguszewski

**Affiliations:** 1 Centro de Obesidade São Paulo SP Brasil Centro de Obesidade, Hospital 9 de Julho, São Paulo, SP, Brasil; 2 Obesidade e Síndrome Metabólica São Paulo SP Brasil Grupo de Obesidade e Síndrome Metabólica, Departamento de Endocrinologia e Metabolismo, Universidade de São Paulo, São Paulo, SP, Brasil; 3 Centro de Diabetes de Belo Horizonte Belo Horizonte MG Brasil Centro de Diabetes de Belo Horizonte, Belo Horizonte, MG, Brasil; 4 Instituto Estadual de Diabetes Rio de Janeiro RJ Brasil Instituto Estadual de Diabetes e Endocrinologia Luiz Capriglione, Rio de Janeiro, RJ, Brasil; 5 Grupo de Diabetes Departamento de Endocrinologia São Paulo SP Brasil Grupo de Diabetes, Departamento de Endocrinologia, Universidade de São Paulo, São Paulo, SP, Brasil; 6 ConscienHealth Pittsburgh PA USA ConscienHealth, Pittsburgh, PA, USA; 7 Associação Brasileira para o Estudo da Obesidade e Síndrome Metabólica São Paulo SP Brasil Presidente Associação Brasileira para o Estudo da Obesidade e Síndrome Metabólica (Abeso), São Paulo, SP, Brasil; 8 Serviço de Endocrinologia e Metabologia Universidade Federal do Paraná Departamento de Medicina Interna Curitiba PR Brasil Serviço de Endocrinologia e Metabologia (SEMPR), Departamento de Medicina Interna, Universidade Federal do Paraná, Curitiba, PR, Brasil; 9 Sociedade Brasileira de Endocrinologia e Metabolismo São Paulo SP Brasil Presidente da Sociedade Brasileira de Endocrinologia e Metabolismo (SBEM), São Paulo, SP, Brasil

## Abstract

Obesity is a chronic disease associated with impaired physical and mental health. A widespread view in the treatment of obesity is that the goal is to normalize the individual's body mass index (BMI). However, a modest weight loss (usually above 5%) is already associated with clinical improvement, while weight losses of 10%-15% bring even further benefits, independent from the final BMI. The percentage of weight reduction is accepted as a treatment goal since a greater decrease in weight is frequently difficult to achieve due to metabolic adaptation along with environmental and lifestyle factors. In this document, the Brazilian Society of Endocrinology and Metabolism (SBEM) and the Brazilian Society for the Study of Obesity and Metabolic Syndrome (ABESO) propose a new obesity classification based on the maximum weight attained in life (MWAL). In this classification, individuals losing a specific proportion of weight are classified as having “reduced” or “controlled” obesity. This simple classification – which is not intended to replace others but to serve as an adjuvant tool – could help disseminate the concept of clinical benefits derived from modest weight loss, allowing individuals with obesity and their health care professionals to focus on strategies for weight maintenance instead of further weight reduction. In future studies, this proposed classification can also be an important tool to evaluate possible differences in therapeutic outcomes between individuals with similar BMIs but different weight trajectories.

## INTRODUCTION

Obesity is a chronic and recurrent disease associated with several complications, which in turn cause and aggravate other acute and chronic diseases and reduce life expectancy ( 1 – 3 ). Although highly stigmatized and perceived by many as a “lifestyle choice” easily treatable by changes in behavior, obesity is instead associated with considerably high rates of treatment failure and a progressive course across life ( 3 , 4 ). Obesity has a complex physiopathology, in which attempts to lose weight are counterbalanced by reduced energy expenditure and increased hunger and desire to eat mediated by the hypothalamus and brainstem, driving weight regain ( 5 – 8 ). The observation of these mechanisms led to the hypothesis that the body must defend a weight “set point.” Despite many knowledge gaps on how this set point changes upward throughout life and whether the set point would be more a range than a fixed value ( 5 , 9 ), clinical evidence suggests that attempts to lose weight are generally counterbalanced by a trend toward weight regain after a weight-loss intervention ( 3 , 5 ). Moreover, there is no evidence that this set point resets downward; instead, the available literature shows that the metabolic adaptation remains the same or decreases in the long term ( 10 – 12 ).

“Resolution” of obesity is rarely achieved with clinical treatment. Indeed, a substantial number of studies have clearly shown that clinically achievable weight loss reduces health risks independent from the final weight ( 13 – 15 ). Several guidelines worldwide recommend a weight loss of 5%-10% ( 3 , 16 – 19 ), but no guideline, as far as we know, has proposed to identify and classify individuals who had lost weight in the past and were able to maintain the loss. This is a critical issue, considering that clinical practitioners usually recommend further weight loss (often clinically difficult to achieve) to individuals who remain with increased BMI after weight loss. These individuals are considered “high risk” by health insurance companies, perceive themselves as having increased risk for several diseases, and do not focus on weight maintenance, increasing the odds of weight regain and yo-yo dieting ( 4 , 20 – 22 ).

In this document, the Brazilian Society of Endocrinology and Metabolism ( *Sociedade Brasileira de Endocrinologia e Metabologia* – SBEM) and the Brazilian Association for the Study of Obesity and Metabolic Syndrome ( *Associação Brasileira de Estudo da Obesidade e Síndrome Metabólica* – ABESO) propose a classification for obesity using the maximum weight attained in life (MWAL, or highest-ever weight) and the percentage of weight loss achieved to guide clinical management and individual decisions. This concept could also be useful in clinical trials since individuals with obesity with different weight trajectories can have different outcomes ( 6 , 23 , 24 ). This proposed classification can also help further disseminate the simple but underappreciated concept of health benefits from clinically achievable weight loss and highlight the importance of obtaining an accurate history of the individual's weight trajectory during evaluation and management of obesity and related disorders ( 25 ). Importantly, the aim of this proposed classification is not to replace traditional and consolidated classifications but rather be an adjuvant tool to guide clinical treatment and help interpret the findings of clinical research and interventions in the field of obesity. This classification can be further improved in the future and be validated in observational and intervention studies. Once published, this classification will be tested in different clinical scenarios to evaluate its usefulness before its widespread use.

### Classifications of obesity

Obesity, recognized by several entities as a chronic and progressive disease, has been defined by the World Health Organization as an “excess fat accumulation that impairs health” ( 26 , 27 ). Excess fat accumulation as a concept seems simple, but its definition is not straightforward. The BMI, calculated as weight divided by squared height, is the most common and accepted tool to diagnose overweight or obesity but has several caveats and large interindividual risk variability ( 3 , 28 , 29 ). Although useful for epidemiological data, BMI often fails to determine the individual's risks in a clinical setting ( 3 , 30 – 32 ). Differences in body composition (fat mass and fat-free mass) and fat distribution are some of the factors that reduce the diagnostic accuracy of the BMI in assessing health risks at individual levels ( 3 , 18 , 28 , 30 ).

Waist circumference (WC) has been proposed as a complementary tool for evaluation of risks associated with obesity, and its importance as a marker of cardiometabolic health independent from BMI has been shown in many studies ( 29 , 31 , 33 – 35 ). Increased WC is an undeniably excellent marker of cardiometabolic status in individuals with normal weight or overweight, but in individuals with higher BMI, WC measurements are less useful in identifying whether the excess fat occurs predominantly due to subcutaneous or visceral abdominal fat ( 31 , 35 , 36 ). High interindividual and intraindividual variability in WC measurement is another limitation of this tool ( 29 , 37 , 38 ).

Other available means to evaluate body composition and distribution that can be useful in clinical practice or research include bioimpedance analysis, dual-energy x-ray absorptiometry, and computed tomography scanning, although these tools are rarely used for diagnosis or management of obesity ( 39 – 41 ).

Considering the pandemic nature of obesity and the fact that BMI is not a good predictor of individual health status, the strategy of defining a subclassification for obesity that could provide priority treatment access for high-risk individuals seems well-founded ( 32 , 42 ). Several ways to classify obesity as metabolically “healthy” or “unhealthy” have been proposed. However, widespread use of this classification has been curbed by controversies surrounding the criteria defining metabolic health, cutoff levels, and inclusion of more complex measures of disease (such as insulin resistance or hepatic fat) ( 43 , 44 ).

A classification of obesity based on the presence of comorbidities and disabilities as a staging system (similar to the classifications used in oncology) has also been proposed, for example, the Edmonton Obesity Staging System ( 18 , 32 , 45 ). This system is simple and useful to evaluate the risks and benefits of different obesity treatments but has some limitations as, for example, the parameter of “psychological burden” included in the classification cannot be objectively defined. Some professional associations have suggested that the term “obesity” should be changed and that the classification of excess fat impairing health should receive different terminologies – such as adiposopathy or adiposity-based chronic disease (ABCD) – but these recommendations have only been used in limited settings ( 32 , 46 ).

### Weight loss of 5%, 10%, 15% or more and reduced risks

Even modest weight losses are associated with health and quality of life benefits ( 14 ). Several guidelines on the clinical treatment of obesity indicate that weight losses of 5%-10% are clinically significant and recommend this range as a treatment target ( 4 , 16 – 19 ). Weight losses of 3% or less are associated with benefits on fertility and glucose levels ( 14 , 47 – 49 ). Some authors have suggested that a weight loss of 3% can be associated with a decreased likelihood of complications from infectious diseases, including COVID-19 ( 50 , 51 ). When above 5%, the weight loss has significant effects on metabolic markers (such as HDL-cholesterol) ( 52 ), depression, joint pain, and sexual function ( 14 , 53 – 56 ). A weight loss goal of 7% has been associated with a lower risk of type 2 diabetes in the Diabetes Prevention Program (DPP) trial, in which each kilogram lost was associated with a nearly 16% reduction in diabetes risk ( 48 , 49 ). Weight losses above 10% have important effects on steatohepatitis ( 14 , 57 , 58 ). A *post hoc* analysis of the LOOK AHEAD trial evaluating intensive lifestyle modification over 9 years in individuals with type 2 diabetes has found that treatment responders with a 10% weight loss had a 21% reduction in the primary outcome of cardiovascular events ( 59 ). Additionally, an 11% weight loss has been associated with a nearly 23% reduction in intra-abdominal adipose tissue, confirming that voluntary weight loss has a disproportionally positive effect on ectopic fat deposition, which is associated with atherosclerosis ( 36 , 60 ).

The DiRECT trial evaluated diabetes remission in individuals with a recent diagnosis of type 2 diabetes and reported that weight losses of 10 and 15 kg (about 10% and 15%, respectively, of the individuals' initial weight) were associated with rates of diabetes remission of 57% and 86%, respectively ( 61 ). A similar study from the same trial in which the mean weight loss was 14% showed normalization of liver fat in individuals achieving diabetes remission and a reduction in the predicted cardiovascular risk score (QRISK) from 23% to 7% ( 62 , 63 ). A recent weight-matched study evaluated individuals undergoing Roux-en-Y gastric bypass or diet-induced weight loss who presented a mean weight loss of 18% and confirmed that the metabolic benefits were induced mainly by weight loss, suggesting dramatic positive effects with the achieved weight loss, even though the final BMI in both groups remained above 35 kg/m^2^ ( 64 ). The same group that conducted the study has also demonstrated clearly reduced inflammation with weight loss above 16% ( 60 ). This is the same proportion of weight loss achieved after 10 years by individuals in the vertical-banded gastroplasty arm in the Swedish Obese Subjects (SOS) study; these individuals comprised 70% of the entire cohort and were not expected to present metabolic effects beyond the weight loss, due to the nature of the procedure ( 14 , 65 ). Even though the SOS study was not powered to compare bariatric procedures, the overall cohort had a substantial decrease in overall mortality and a life expectancy increase lasting at least 24 years ( 13 , 66 ). These individuals had a final BMI of approximately 35 kg/m^2^, indicating that two individuals with the same weight but different weight trajectories can exhibit entirely different overall risks ( 63 ). Questions remain regarding the existence of a specific threshold below which the risks decrease or whether progressive weight loss is associated with a proportional decrease in risks. Weight losses of 16%-20% or more are rarely achievable in the long term with currently available clinical therapies ( 66 ). However, this scenario can potentially change with the development of new and more efficacious antiobesity drugs ( 67 , 68 ). As such, clinically achievable weight loss has been clearly recommended as the goal in obesity treatment, and proposals have emerged indicating that the achievement of metabolic health in individuals with obesity is the “low-hanging fruit” for treatment ( 69 ). A classification for individuals who are able to achieve such goals is imperative.

### Proposed classification based on the weight trajectory and the maximum weight achieved in life

This new classification ( Table 1 ), deliberated by a working group from both Brazilian medical societies, identify individuals based on weight trajectory during clinical treatments for obesity (including non-pharmacological and pharmacological treatments, as well as therapies using non-surgical devices) and is intended for adults aged 18 to 65 years and with BMI values between 30 and 50 kg/m^2^. The classification does not apply to individuals with end-stage diseases due to lack of benefits from weight loss in these circumstances. It also does not apply to individuals using corticosteroids chronically or intermittently or with endogenously increased cortisol levels due to Cushing's syndrome, or those using other short-term drugs leading to weight gain. We acknowledge that many proposals for reclassification of obesity advocate against using BMI as the only diagnostic criteria, but BMI is the single most common starting point. Therefore, we incorporated BMI into this proposed classification to avoid confusion or complexities at the moment.

**Table 1:** Proposed classification of “reduced” and “controlled” obesity based on maximum body mass index (BMI)

Maximum BMI	Unchanged*	Reduced*	Controlled*
30-40 kg/m^2^	<5%	5-9.9%	>10%
40-50 kg/m^2^	<10%	10-14.9%	>15%

In this proposed classification, the patients should be asked in their first visit about their MWAL (excluding weight recorded during pregnancy). This value should be considered for the primary diagnosis based on the original classification of obesity (Class I, 30.0-34.9 kg/m^2^; Class II, 35.0-39.9 kg/m^2^) followed by the terms “unchanged” (if close to the MWAL), “reduced” (if 5%-10% of weight loss is achieved), or “controlled” (if at least 10% of weight loss is achieved). The percentage of weight loss (by 5% decrements) should also be identified. For individuals with BMI values between 40-50 kg/m^2^, we propose that the term “controlled” should be applied if the weight loss achieved is above 15%, “reduced” if between 10%-15%, and “unchanged” if less than 10%. Below are two examples from Table 2 .

**Table 2:** Newly proposed classification based on two clinical cases

**Case 1:** A 55-year-old man with a maximum weight achieved in life (MWAL) 2 years earlier of 118 kg and a height of 175 cm (BMI of 35.9 kg/m^2^).

Hypothetical scenarios	Weight (kg)	BMI (kg/m^2^)	Percentage of weight loss based on MWAL	Newly proposed classification	Traditional classification
1A	115	38.5	2.5%	Class II obesity (unchanged)	Class II obesity
1B	108	35.2	8.4%	Class II obesity (5% reduced)	Class II obesity
1C	103	33.6	12.7%	Class II obesity (10% controlled)	Class I obesity
1D	98	32.0	16.9%	Class II obesity (15% controlled)	Class I obesity

**Case 2:** A 40-year-old woman with an MWAL of 100 kg (6 months earlier) and a height of 156 cm (BMI 41 kgm^2^).					
Hypothetical scenarios	Weight (kg)	BMI (kg/m^2^)	Percentage of weight loss based on MWAL	Newly proposed classification	Traditional classification
2A	98	41	2%	Class III obesity (unchanged)	Class III obesity
2B	94	39.1	6%	Class III obesity (unchanged)	Class II obesity
2C	89	37	11%	Class III obesity (10% reduced)	Class II obesity
2D	82	34.1	18%	Class III obesity (15% controlled)	Class I obesity

In Case 1, a man with a height of 175 cm and an MWAL of 118 kg has a BMI of 38.5 kg/m^2^ and Class II obesity (unchanged). If he lost 10 kg (8.4% of his maximum weight), he would remain in the Class II obesity category but would be considered as having “reduced obesity” in this newly proposed classification since he could derive clinical benefits from his weight loss. Had only his BMI values been considered, no change would have occurred, and his weight loss would be considered insufficient or his treatment a failure. If he lost 15 kg (12.7% of his MWAL), he would still be categorized as having Class II obesity based on his MWAL and would be considered “controlled” based on his weight loss of 12.7%. Thus, the decision to lose more weight should be analyzed individually based on the patients' overall health and metabolic status (and not solely on BMI values). Should the patient lose even more weight (20 kg), he would very likely show clinical improvement but would remain in the same category in the original classification. In the new proposed classification, he would be considered as having Class II obesity (15% controlled).

In Case 2, a woman with a height of 156 cm and an MWAL of 100 kg has a BMI of 41 kg/m^2^ and Class III obesity. If she lost 6 kg, she would be reclassified as having Class II obesity based on her BMI but would still be considered to have Class III obesity (unchanged) according to the proposed classification since she did not achieve a minimum of 10% to be considered “reduced.” If she lost more than 10 kg (11 kg in Scenario 2C in Table 2 ), she would be categorized as having Class III obesity (10% reduced), and if she lost 18 kg, she would still be categorized as having Class III obesity (15% controlled) in the proposed classification.

If an individual lost a substantial amount of weight (as in Scenarios 1D or 2D) and is not able to lose more weight, his or her goal would be to maintain weight instead of losing more weight, which would probably happen had only the new BMI been considered.

For regions or ethnic groups defining criteria for overweight and obesity by different BMI cutoff values, the classification can follow the specific local and ethnic criteria. This proposed classification can also help individuals understand that obesity is a chronic disease and, regardless of their current weight status, their biology is driven toward weight regain. It also includes the MWAL as important information to be collected in the patient's medical history.

Of note, this document is not intended to determine the criteria for success after bariatric procedures, and the proposed terms “reduced” and “controlled” are applicable to the success of clinical but not surgical treatments. Still, we recognize that the idea of classifying obesity based on MWAL and percentage of achieved weight loss could be useful in individuals undergoing bariatric surgery and be applied to guide clinical decisions. However, some adjustments to the classification would be necessary, including the differentiation of insufficient weight loss and long-term weight regain after the bariatric procedure.

### Special situations

#### Pregnancy and lactation

Weight gain is expected to occur during pregnancy. Although some women gain more weight than expected and struggle to lose the gained weight in the postpartum period, the weight generally decreases naturally during and after lactation ( 70 – 73 ). The maximum weight achieved in pregnancy should be accurately written in the patient's medical history but should not be used for the classification proposed in this document. Instead, the MWAL should consider nonpregnant conditions. If the MWAL was achieved after pregnancy, the stable weight reached after the end of the lactation should be considered as the MWAL.

#### Involuntary weight loss

Several life-threatening diseases lead to involuntary weight loss and cachexia; these conditions are the source of important bias in epidemiological studies evaluating the relationship between weight loss and health status ( 25 , 74 – 76 ). Since long-term voluntary weight loss and maintenance are hard to achieve, most individuals losing substantial weight in large observational datasets are likely to be those with severe diseases ( 77 , 78 ). If the weight loss achieved clinically is suspected of having occurred involuntarily, the classification proposed in this document should not be used, and an investigation of the possible causes for the weight loss is warranted.

#### Individuals with end-stage chronic diseases

This proposed classification does not apply to individuals with end-stage diseases ( *e.g.* , patients with chronic renal failure undergoing dialysis, heart failure NYHA classes III or IV, cirrhosis, or metastatic cancers with reduced overall survival, among others). For individuals with these conditions – classified as stage 4 in the Edmonton Score – palliative measures are more important than weight loss ( 45 ).

#### Older age

We chose to limit this new classification to adults younger than 65 years. Altered body composition – particularly loss of lean tissue (mainly muscle mass) and increased body fat – become more evident with age and can have profound metabolic effects ( 79 – 81 ). With age, BMI values usually stabilize or reduce, but visceral fat and intramyocellular fat increase ( 79 , 82 ). As such, the diagnosis of obesity based only on body weight is imperfect, so body composition data should be considered in the diagnosis; this has resulted in different cutoff values for healthy BMI proposed for this age group ( 83 – 85 ). At the same time, the possibility of concomitant diseases leading to involuntary weight loss increases with age ( 76 , 80 , 86 ). Still, individuals with obesity losing weight voluntarily can derive clinical benefits, but caution regarding sarcopenia and osteoporosis associated with the weight loss should be exercised ( 89 , 87 – 90 ).

#### Children and adolescents

This new classification is not intended for individuals younger than 18 years. In this population, obesity should be diagnosed based on BMI Z-scores according to age, weight, and height ( 91 ). Children and adolescents with obesity have higher odds of becoming adults with obesity, and many of the concepts discussed previously in this document may apply to this younger population ( 92 , 93 ). A classification dedicated to children and adolescents with obesity should consider the social burden and stigma of this diagnosis in young individuals.

#### Exogenous or endogenous hypercortisolemia

Chronic and recurrent use of corticosteroids can lead to weight fluctuations, hindering proper interpretation of the MWAL and identification of the weight as being voluntary or involuntary ( 94 ). Cushing's syndrome, treated or untreated, can also affect body weight regulation ( 95 ). As such, the proposed classification should not be used in individuals receiving active treatment for Cushing's syndrome or using corticosteroids intermittently. In individuals with long-term remission from Cushing's syndrome or who were chronically treated with corticosteroids in the past but are no longer receiving such treatment, the proposed criteria should be used with caution, and the MWAL should be considered after the treatment period.

#### Acute and reversible situations that could lead to weight gain

Overfeeding studies have shown that the body presents adaptive responses that curb weight gain in the long term, such as those observed with caloric restriction but in the opposite direction. During short-term overfeeding ( *e.g.* , occurring during holidays or vacations), any weight gain is at least partially reversible after the individual returns to routine daily life ( 96 – 98 ). Therefore, if the MWAL was achieved during a very short period of time, the MWAL should be defined as the maximum stable weight that occurred during a longer period, which has been arbitrarily defined in this document as at least 3 consecutive months. Similarly, if the individual gained weight using obesogenic medications – such as antidepressants and antipsychotics – for a very short period, the weight assessed at that moment should not be considered as the MWAL ( 99 ) since weight loss is common after these drugs are withdrawn. However, if these drugs are used during a longer period ( *e.g.* , more than 3 months) and have a substantial impact on the individual's weight, and if the MWAL was reached in this setting, this MWAL should be considered even if the drugs are withdrawn later, since chronic use of these medications is a risk factor for obesity ( 100 , 101 ).

#### Use of antidiabetic agents leading to weight gain

Type 2 diabetes is intrinsically related to weight gain and obesity. In our view, the concept of “controlled” obesity proposed in this new classification is extremely useful in patients with this disease, particularly in the first 6-8 years after diagnosis, when significant weight loss can change the natural course of the disease and even lead to diabetes remission ( 52 , 61 , 63 , 66 ). However, many antidiabetic agents are associated with weight gain ( *e.g.* , insulin, sulfonylureas, glinides, and thiazolidinediones) ( 99 , 102 , 103 ). If these medications are used chronically, no changes should be made to the classification, and the concept of “controlled” obesity can support the use of antidiabetic agents with a more favorable effect on weight ( 102 ). Weight gained after short-term intensification of insulin ( *e.g.* , during hospitalization, acute illness, or related to glucotoxicity) that is further discontinued or reduced in dose should not be considered the MWAL.

#### Smoking cessation and relapse

Smoking cessation is associated with weight gain in most individuals ( 104 – 106 ). The MWAL can be considered when recorded after smoking cessation, but if smoking relapses and the individual loses weight, this “tobacco-induced” weight loss should be seen as involuntary and not considered to classify the individual as having “reduced” or “controlled” obesity.

#### Overweight individuals

Overweight is associated with increased health risks. Several guidelines suggest weight loss for individuals with overweight, particularly when associated with comorbidities ( 1 , 16 – 19 ). We believe that the idea of “reduced” and “controlled” overweight is valid in individuals with overweight the same way that it is applied for obesity, particularly in patients with comorbidities. However, we emphasize that this proposed classification applies to individuals with obesity defined as a BMI above 30 kg/m^2^.

#### Metabolically unhealthy, normal-weight individuals

Even with a “normal” BMI, some individuals have metabolic abnormalities related to excess adiposity with abnormal distribution and probably benefit from losing weight below an individual threshold ( 107 , 108 ). The ABCD classification has been proposed for these individuals since it focuses on metabolic abnormalities rather than weight, but several controversies remain in terms of the criteria that should be used to consider them as metabolically unhealthy ( 46 ). As described for individuals who are overweight, the proposed classification does not apply for individuals who have normal weight but are metabolically unhealthy.

#### Class IV and V obesity (body mass index above 50 kg/m^2^)

Limited quality data exist on weight loss outcomes in individuals with BMI above 50 kg/m^2^, and it is unclear whether the weight loss percentages used for individuals with BMI 30-50 kg/m^2^ should be applied to consider these individuals “controlled.” Consequently, we decided against proposing specific weight loss thresholds for individuals with BMI values above 50 kg/m^2^, for whom the burden of obesity is extreme and clinical treatments have limited benefits ( 109 , 110 ).

### Strengths and limitations

The main strength of this simple new classification is the emphasis on the achievement of weight loss goals with clinical treatment, as defined in several guidelines. This proposed classification yields a more comprehensive view of the individual's weight status and establishes future objectives for patients and health care providers. We believe that this simple and ready-to-use classification can help reduce the stigma of clinical obesity treatment, improve long-term adherence to obesity therapy, and facilitate the understanding that obesity is a chronic and recurrent disease. The only information that must be reliably obtained is the individual's MWAL, which has previously been proposed as important information in the clinical setting ( 23 , 25 , 111 ).

The stigma of obesity – unfortunately very common in health care and across society – is associated with poorer health outcomes and further weight gain ( 112 , 113 ). With this proposed classification being implemented, future studies can be performed to evaluate its impact on weight stigma. However, we believe that this impact will be positive since the classification will enable health care providers to understand – even if partially – the complex regulation of body weight and the benefits of modest weight losses. This proposed classification can also help patients and health care providers in discussing more realistic goals and guide health care professionals not involved in the management of obesity in referring patients for proper care, using it as an adjuvant to other measurements, classifications, and clinical findings. Before widespread implementation of this classification, we plan to conduct questionnaire studies to inquire individuals living with obesity whether they believe this classification could help treat obesity and reduce the stigma of the disease.

Future studies are required to validate this classification for risk stratification and prediction of clinical outcomes. Other classifications ( *e.g.* , the Edmonton Obesity Staging System) were first proposed and then validated in epidemiological studies. We believe the same can be done with our classification ( 42 , 45 , 114 ). This proposed classification could also be useful in interventional obesity studies. Generally, clinical trials do not include these simple data that might influence outcomes ( 25 ). Individuals near their MWALs are expected to lose more weight in response to an intervention compared with individuals with reduced or controlled obesity, who are expected to lose less additional weight ( 6 , 25 ). If a trial enrolls a large number of individuals with reduced or controlled obesity, the impact of the intervention may be underestimated. The same applies to experimental studies since cerebral or hormonal responses to overfeeding, underfeeding, or different food stimulation differ according to the individual's weight-reduced status, which can lead to biased interpretations of the results ( 115 ).

The concept of controlled disease is not new. Indeed, this concept is widely used in individuals with diabetes. Individuals with diabetes with glycated hemoglobin (HbA1c) levels lower than 7.0% can be considered “well controlled” despite this level being above normal ( 116 ). This definition of optimal control is based on several observational and randomized controlled trials showing that a reduction in HbA1c level is associated with improved diabetes outcomes ( 117 , 118 ). Compared with diabetes, the evidence is weaker for obesity due to fewer clinical studies, as previously discussed, but randomized controlled trials have shown that a 5%-15% weight loss is associated with reduced morbidity ( 14 , 48 , 49 , 52 – 59 , 61 , 63 ). Still using diabetes as an example of a disease related to obesity, evidence shows that weight loss reduces the incidence and increases the odds of remission of this disease ( 48 , 49 , 61 ), but proposals on how to record this information in the long term are lacking. As such, the MWAL and the percentage of weight loss can also be used as a simple, objective, and continuous target of disease control in obesity ( 15 ).

This proposed classification also has several limitations, as listed below:

First, this classification relies on self-reported MWAL, which can be subjected to recall bias ( 119 , 120 ). Also, it has been suggested that the weight set point may be a range instead of a fixed value. Moreover, periods of transient overfeeding may lead to acute and (at least partially) reversible weight gain ( 9 , 97 , 121 ). Therefore, it is advisable to consider as the MWAL a stable weight maintained for more than 3 months, although we acknowledge that this period is arbitrary. Development of biomarkers for use as more objective parameters of reduced obesity status would be desirable. Nonetheless, the spread of the concept of MWAL as an important measuring tool could help people living with obesity and health professionals to value this information and reduce the bias of recalling previous weight.

Second, the proposed classification has the known limitations of the use of BMI in clinical practice. As mentioned before, BMI values can be a poor marker of individual health, and variations in body composition and distribution can impact their interpretation ( 28 ). However, this classification relies on more than just BMI, as it also considers the weight history.

Third, the classification adopts only weight loss as a marker of obesity control. The goal of obesity treatment is to improve health and quality of life, and similar weight losses can have a diverse clinical impact on different individuals depending on baseline health status and overall individual conditions ( 3 , 18 ). We acknowledge that for an individual with obesity to be considered “controlled,” the weight loss achieved would have to provide evident clinical benefits such as improvement in metabolic markers, physical functioning, or mental health. However, we decided against adding clinical markers to the classification at the moment to keep it simple for clinicians. Extensive discussions in the literature debate the use of criteria to identify metabolically healthy obesity ( 44 ) and whether this term should even be used ( 44 , 122 ). Additionally, it is difficult to objectively measure without using questionnaires the mechanical and mental health benefits of weight loss. Additionally, the impact of weight loss on mental health has not been broadly evaluated ( 123 ), and any classification based on clinical improvement could be subjective and complicate its implementation. We are aware that an “advanced classification” considering both the weight loss achieved and the clinical benefits could be more precise – albeit less practical – in identifying “controlled” obesity. A future step could be a combination of this proposed classification and an improvement in the Edmonton Obesity Staging System ( 45 ) ( *e.g.* , from EOSS stage 2 to stage 1) or an individual change from a well-defined classification of metabolically unhealthy obesity to metabolically healthy obesity, as proposed by Stefan and cols. ( 69 ).

Fourth, this classification is not a guideline, as discussed above. An individual considered to have controlled obesity could derive benefits from losing further weight or be a candidate for bariatric surgery. Likewise, individuals with reduced (or unchanged) obesity might not need to lose more weight if they have a low overall burden from their high BMI. This classification is rather intended to provide important information for discussion with the patient and, as any other classification, should be considered in the overall context of the patient's health and long-term goals.

Fifth, a wide range of individuals were not included in this proposed classification due to the reasons pointed out above, but their weight trajectories could also be used as a management guide. As such, we see the MWAL as almost a “vital sign” to be asked at every clinical history taking, even if this proposed classification is not applicable to the individual.

Finally, our classification has not been validated and lacks direct evidence showing that a “reduced” or “controlled” obesity status is associated with reduced hard outcomes. At present, the evidence is indirect and based on reduced cardiovascular risk factors observed in mechanistic studies, subanalyses of clinical trials (such as the *post hoc* analysis of the LOOK AHEAD trial), and after bariatric surgery, in which the magnitude of the weight loss is usually higher. The decision of adopting a weight loss of 15% as the criteria for controlled obesity in individuals with a BMI above 40 kg/m^2^ derived mainly from indirect data from the gastric banding and vertical-banded gastroplasty arms of the SOS study ( 65 ). This indirect evidence from surgical studies does not rule out the possibility of weight-independent effects ( 13 , 14 , 59 , 60 , 65 ), and the SOS study was not powered to evaluate differences between procedures. As such, the evidence for the proposed thresholds of “reduced” and “controlled” obesity is weak. Since studies have rarely used the MWAL, we cannot exclude the possibility that a large proportion of the individuals in clinical trials already had (at least slightly) a weight reduction and, as such, the benefits from a modest weight loss ( *e.g.* , 5%-10%) from the MWAL could have a lower clinical significance. We are aware of these limitations, but we believe that this classification could help future trials answer questions by objectively categorizing individuals into groups according to their previous weight trajectories. Since weight history is rarely recorded in medical charts, this proposed classification could not be validated before being implemented. A recent observational study of risk prediction evaluated patients' weight history based on data from medical records and a follow-up of 6 years. Aligned with our proposal, individuals with more than 15% of weight loss developed cardiometabolic outcomes later than those with less than 7% of weight loss ( 124 ). The same authors also found similar results regarding osteoarthritis and health care utilization ( 125 ). Nonetheless, no information was provided about MWAL, and their classification was different from ours. A more unified or known definition could facilitate the research in the field. Another less conclusive example is a recent prospective cohort study evaluating BMI and prognosis of COVID-19, in which the authors evaluated whether prior weight loss was protective against severe COVID-19, but the analysis could not be performed because the weight changes were poorly reported ( 126 ). If the importance of recording weight history in medical charts is not emphasized and the concept of “reduced” and “controlled” obesity is not widely known, these issues will probably never be adequately addressed. Even if this proposed classification is not readily applied before further validation, it still can be useful as a “call for action” for health care professionals to record the patients' MWAL and understand its usefulness.

In conclusions, this document proposes a new and relatively simple classification of obesity for adults aged 18-65 years based on the percentage of weight loss from the MWAL and using the terms “reduced” and “controlled” obesity. This proposed classification intends not to replace other classifications but rather serve as an adjuvant tool. This classification could have practical implications on obesity care and, after validation in future studies, could be improved with new data and input, especially if combined with clinical markers. Both SBEM and ABESO intend to validate this classification before it is widely utilized but believe that it could be a very useful tool to help clinical decisions and reduce the stigma of obesity. This classification could also be useful in future epidemiological, mechanistic, and interventional studies.
